# Serum paraoxonase 1 and 3 activities in benign and malignant diseases of the prostate and changes in levels following robotic-assisted laparoscopic radical prostatectomy

**DOI:** 10.3906/sag-2004-353

**Published:** 2020-12-17

**Authors:** Fevzi BEDİR, Hüseyin KOCATÜRK, Mehmet Sefa ALTAY, Engin ŞEBİN, Banu BEDİR

**Affiliations:** 1 Department of Urology, Health Sciences University, Erzurum Regional Training and Research Hospital, Erzurum Turkey; 2 Department of Biochemistry, Health Sciences University, Erzurum Regional Training and Research Hospital, Erzurum Turkey; 3 Department of Public Health, Aziziye District Health Directorate, Erzurum Turkey

**Keywords:** Paraoxonase 1, paraoxonase 3, malondialdehyde, prostate cancer, benign prostatic hyperplasia

## Abstract

**Background/aim:**

This study aimed to examine serum paraoxonase 1 and 3 (PON1 and PON3) activities in benign and malignant diseases of the prostate, to determine lipid profile and malondialdehyde (MDA) levels, and to investigate changes in levels following robotic-assisted laparoscopic radical prostatectomy (RALRP).

**Materials and methods:**

A total of 137 patients, including a control group, were enrolled in the study and assigned into four groups. Group 1 (n = 33) consisted of patients previously undergoing RALRP with no recurrence, group 2 (n = 36) consisted of patients diagnosed with prostate cancer (PCa) and undergoing RALRP, and group 3 (n = 34) consisted of patients diagnosed with benign prostatic hyperplasia. The control group (n = 34) consisted of healthy individuals. Serum low-density lipoprotein (LDL), high-density lipoprotein (HDL), triglyceride, cholesterol, prostate-specific antigen (PSA), PON1, PON3, and MDA values were measured. In addition, group 2 MDA, PON1, PON3, and PON1/HDL levels were investigated preoperatively and at the first month postoperatively.

**Results:**

Significant changes were found in PON1, PON3, and MDA levels. PON1 and PON3 levels decreased significantly in patients with PCa, while MDA levels increased. PON1 and PON3 increased postoperatively in the PCa group, while MDA decreased. BPH group PON1, PON3, and MDA levels were higher than those of the control group.

**Conclusion:**

An increase in free oxygen radicals in the body or a decrease in endogenous antioxidant enzyme levels can result in malignant and benign diseases of the prostate. Surgical excision of malignant tissue in PCa causes a decrease in oxidative stress.

## 1. Introduction

Prostate cancer (PCa) is the most common type of solid cancer in developed countries and the second most common form of cancer, after lung cancer, in men worldwide [1]. The incidence of PCa increases with age and it is most prevalent after the age of 65 [2]. PCa risk factors include hormonal and genetic factors, black ethnicity, poor socioeconomic status, a fat-rich diet, and infections [3]. Studies have shown that oxidative stress of various endogenous and exogenous origins increases the risk of PCa [4].

Free oxygen radicals (FORs) and lipid peroxidation are implicated in the etiopathogenesis of several diseases [5]. FORs react with such important compounds as lipids, protein, DNA, and carbohydrates to compromise their structures. FORs and other free radicals in biological systems are one of the most important causes of oxidative stress [6]. In addition to DNA damage and associated tumor oncogenesis, this also results in the development of cancer and metastasis by affecting such cellular functions as cell proliferation, remodeling, and apoptosis [7].

Malondialdehyde (MDA) is the end-product of the peroxidation of polyunsaturated fatty acids and circulating levels increase in line with FORs. These can be observed as a marker in cancer patients. Cell damage leading to carcinogenic effects is known to occur with an increase in MDA [7].

Endogenous and exogenous antioxidants reduce tumor development by balancing the adverse effect established by FORs. The endogenous antioxidant enzyme paraoxonase (PON) complex occupies an important place among these [8]. Three members of the PON gene family are known (PON1, PON2, and PON3). PON1 is present in the liver, PON3 in the liver and kidney, and PON2 in various tissues including the kidney, liver, lungs, small bowel, placenta, spleen, gut, and testes [9]. The enzymes PON1 and PON3 are associated with high-density lipoprotein (HDL) and exhibit antioxidant properties. All PON enzymes in circulation hydrolyze peroxidase, lactonase, arylesterase activities, and oxidized phospholipids. PON1 activity is regulated genetically and, through diet, prevents atherosclerosis by reducing macrophage foam cell formation by protecting against low-density lipoprotein (LDL) oxidation. PON3 exhibits its antioxidant effect by stimulating LDL formation and monocyte chemotactic activity [10].

The present study investigated differences in PON1 and PON3 activities and serum MDA levels and lipid profile levels between patients with Pca, patients with benign prostatic hyperplasia (BPH) having previously undergone RALRP, and a control group.

## 2. Materials and methods

### 2.1. Patient group and study protocol

This study was conducted in line with the ethical standards specified by the Declaration of Helsinki and following receipt of local ethical committee approval (2018/18-169). Following receipt of informed consent, transrectal USG (TRUSG)-guided biopsy was collected from patients presenting to our polyclinic with PSA elevation and/or findings at the rectal examination. Patients pathologically confirmed as PCa or BPH and patients undergoing RALRP with a diagnosis of PCa were included in the study. The control group consisted of healthy individuals with no LUTS symptoms, normal PSA values, and no previous history of infection or drug use for any reason. In addition, patients with previous histories of RALRP and no biochemical recurrence were evaluated as a distinct group. All patients taking part in the study underwent a detailed physical examination by the same urologist. The patients’ age, body mass index (BMI), smoking status and alcohol consumption, LDL, HDL, triglyceride, cholesterol, PSA, PON 1, PON 3, MDA, and PON1/HDL values were investigated.

One hundred and thirty-seven patients, including the control group, were included in the study. Patients were divided into four groups. Group 1 (n = 33) consisted of patients who had previously undergone RALRP with no recurrence. Group 2 (n = 36) consisted of patients diagnosed with PCa and undergoing RALRP. Group 3 (n = 34) consisted of patients diagnosed with BPH. The control group (n = 34) consisted of healthy individuals. Group 2 preoperative, postoperative, and 1st month MDA, PON1, and PON3 levels were also investigated.

### 2.2. Inclusion criteria

Patients with prostate biopsy indication undergoing standard 12-core TRUSG-guided biopsy and diagnosed with PCa or BPH were included in the study. In addition, patients diagnosed with PCa indicated for surgery and undergoing RALRP in our clinic, and patients who had previously undergone RALRP and who had no biochemical recurrence were also enrolled. The control group consisted of healthy individuals.

### 2.3. Exclusion criteria

Patients with a known history of cardiac, renal, hepatic, or endocrinological disease, smokers, and patients consuming alcohol, patients with histories of lipid-lowering, antioxidant, or vitamin medication use, and patients with clinical or radiological suspicion of metastasis were excluded from the study.

### 2.4. Sample collection and laboratory methods

Specimens were placed into tubes without anticoagulant and then stored for 2 h in line with the instructions for the ELISA kits employed. They were then centrifuged for 15 min at 1000 × *g* at 4 °C. The supernatant parts were then transferred into Eppendorf tubes and stored at –80 °C until analysis. MDA was analyzed using Human Malondialdehyde ELISA Kits (Bioassay Technology Laboratory, Cat. No: E1371Hu, Shanghai, China). PON 1 was analyzed using Human Paraoxonase-1 ELISA Kits (Bioassay Technology Laboratory, Cat. No: E2157Hu, Shanghai, China). PON 3 was analyzed using Human Paraoxonase-3 ELISA Kits (Bioassay Technology Laboratory, Cat. No: E2066Hu, Shanghai, China).

### 2.5. Routine parameters

Triglyceride and total cholesterol were measured using kits on an Architectc 16000 (Abbott Diagnostics, Illinois, USA) device. HDL and LDL were measured using kits (Archem Diagnostics, İstanbul, Turkey) on an Architectc 16000 (Abbott Diagnostics, Illinois, USA) device. PSA was measured using Abbott kits on an Abbott Architect i2000 device.

### 2.6. Statistical analysis

Statistical analysis was performed on the Statistical Package for Social Sciences (SPSS) v20 for Windows (IBM Corp., in Armonk, NY, USA) software. Categorical variables were expressed as number and percentage, and numerical variables as mean plus standard deviation. Appropriateness for analysis of numerical variables was assessed using the Kolmogorov–Smirnov test. The Mann–Whitney U and Kruskal–Wallis tests were used to compare numerical variables during the performance of hypotheses. The Bonferroni test, which is one of the posthoc test statistics, was used to determine the source of the significant difference between the groups as a result of the analysis. Wilcoxon test was used to analyze the data of group 2 before and after the surgery. Spearman rank correlation was applied to determine relations between MDA and PON1 and PON3 values. P values ˂ 0.05 were regarded as statistically significant.

## 3. Results

One hundred and thirty-seven participants were included in the study. The mean ages of the patients were 66.27 ± 3.48 in group 1, 65.42 ± 2.96 in group 2, 65.50 ± 2.30 in group 3, and 66.53 ± 1.61 in the control group. No statistically significant difference was determined between the groups (P = 0.61). Various demographic characteristics and biochemical values of the patient groups are shown in Table 1 and Figure.

**Table 1:** Various patient group demographic characteristics and biochemical values.

	Group 1	Group 2	Group 3	Control	
	Median(minimum–maximum)	Median(minimum–maximum)	Median(minimum–maximum)	Median(minimum–maximum)	P*
Age (years)	66.27 (56–70)	65.42 (58–70)	65.50(55–74)	66.53(59–69)	p=0.61
BMI (kg/m2)	27.00 (20.00–50.00)	28.00 (20.00–36.00	26.00 (22.00–38.00)	27.50 (21.00–34.00)	p=0.632
LDL (mg/dL)	122.00 (79.00–205.00)	121.50 (67.00–203.00)	142.00 (55.00–187.00)	120.50 (54.00–202.00)	p=0.098
HDL (mg/dL)	40.54 (28.72–68.42)	47.75 (31.00–87.79)	45.54 (28.57–66.60)	47.02 (22.70–61.83)	P = 0.193
Cholesterol (mg/dL)	201.00 (134.00–276.00)	193.50 (123.00–273.00)	199.50 (132.00–296.00)	191.00 (101.00–286.00)	P = 0.338
Triglyceride (mg/dL)	188.00 (58.00–650.00)	128.50 (66.00–420.00)	154.50 (76.00–340.00)	168.00 (43.00–270.00)	P = 0.144
PSA (ng/mL)	0.007 (0.001–0.07)	7.75 (4.33–24.90)	6.32 (0.98–34.85)	1.49 (0.09–2.82)	P ˂ 0.001
MDA (nmol/mL)	7.92 (2.57–11.21)	9.10 (2.02–11.92)	7.72 (3.23–10.46)	6.42 (0.46–9.99)	P ˂ 0.001
PON1 (mg/L)	118.79 (20.91–952.73)	42.43 (24.22–74.93)	105.47 (24.32–625.05)	81.57 (18.45–1073.02)	P ˂ 0.001
PON 3 (mg/L)	4.52 (1.53–40.23)	3.19 (1.26–5.03)	5.23 (1.83–27.70)	4.75 (1.92–28.13)	P ˂ 0.001
PON1/HDL	3.04 (0.31–22.69)	0.91 (0.42–1.53)	2.21 (0.44–11.11.16)	1.75 (0.42–24.19)	P ˂ 0.001

* Kruskal–Wallis test was used to analyze the data. BMI: Body mass index, LDL: Low-density lipoprotein cholesterol, HDL: High-density lipoprotein cholesterol, PSA: Prostate specific antigen, MDA: Malondialdehyde, PON1: Paraoxonase 1, PON3: Paraoxonase 3, P: level of significance.

**Figure:**
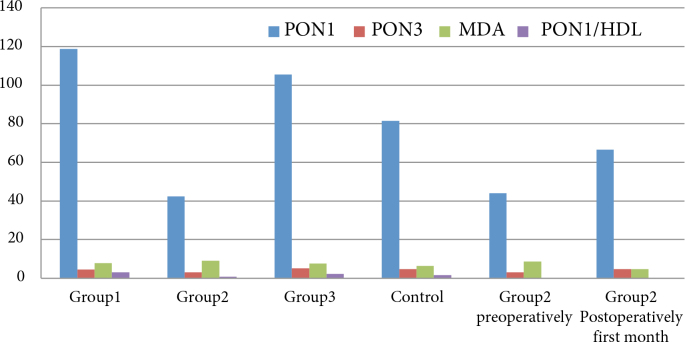
Distributions of PON1, PON3, MDA, and PON1/HDL among the groups. MDA: malondialdehyde; PON1: paraoxonase 1; PON3: paraoxonase 3; PON1/HDL: paraoxonase 1/high-density lipoprotein. Group 1 consisted of patients who had previously undergone RALRP and had no recurrence. Group 2 consisted of patients diagnosed with PCa and undergoing RALRP. Group 3 consisted of patients diagnosed with BPH. The control group consisted of healthy individuals. Group 2 preoperative consisted of patients diagnosed with PCa preoperative; group 2 postoperative consisted of patients diagnosed with PCa 1 month after undergoing RALRP.

Intergroup comparisons revealed statistically significant differences between groups 1 and 2 in terms of PON1, PON3, and MDA (P ˂ 0.001, P ˂ 0.001, and P = 0.045, respectively). No significant difference was observed between groups 1 and 3 in terms of PON1, PON3, or MDA (P = 0.419, P = 0.679, and P = 0.190, respectively). Significant differences were detected between group 1 and the control group in terms of PON1 and MDA (P = 0.010 and P = 0.004, respectively), but not in terms of PON3 (P = 0.590). Significant differences were also observed between groups 2 and 3 and between group 2 and the control group in terms of PON1, PON3, and MDA (P ˂ 0.001 for all). Group 3 and the control group differed significantly only in terms of MDA (P = 0.034).

The median preoperative MDA value of the patients in group 2 was 9.10 (min–max: 2.02–11.93), the median PON1 value was 42.43 (min–max: 24.22–74.93), and the mean PON3 value was 3.19 (min–max: 1.26–5.03). Following the surgery, the median MDA value decreased to 4.85 (min–max: 0.52–9.59), the median PON1 value increased to 65.58 (min–max: 26.39–111.22), and the median PON3 value increased to 4.83 (min–max: 1.73–10.72). The differences between the pre- and postoperative MDA, PON1, and PON3 values in group 2 were all statistically significant (P ˂ 0.001) (Table 2).

**Table 2:** Pre- and postoperative MDA, PON1, and PON3 values of the patients in Group 2.

	Group 2 preoperative	Group 2 postoperative	P
	Median(minimum–maximum)	Median(minimum–maximum)	
MDA (nmol/mL)	9.10 (2.02–11.92)	4.85 (0.52–9.59)	P ˂ 0.001
PON1 (mg/L)	42.43 (24.22–74.93)	65.58 (26.39–111.22)	P ˂ 0.001
PON3 (mg/L)	3.19 (1.26–5.03)	4.83 (1.73–10.72)	P ˂ 0.001

* Wilcoxon test was used to analyze the data. MDA: Malondialdehyde, PON1: Paraoxonase 1, PON3: Paraoxonase 3, P: level of significance.

A weak, negative significant correlation was present between PON1 and PSA as PON1 decreased when PSA increased. A weak, negative correlation was present between PON3 and PSA, with PON3 decreasing as PSA increased. A weak positive correlation was observed between HDL and PON3, with PON3 increasing in line with HDL. No correlation was determined between MDA and PSA, although MDA exhibited a statistically significant positive correlation with age. No significant differences were observed between our study groups in terms of triglyceride, LDL, and HDL.

In order to determine whether or not the decrease in PON1 serum activity caused a decrease in HDL levels or enzyme activity, we calculated the PON/HDL ratio and observed statistically significant differences between groups 1 and 2 (P ˂ 0.001), and between group 1 and the control group (P = 0.008). Significant differences were present between groups 2 and 3 (P ˂ 0.001) and between groups 2 and 4. A powerful positive correlation was determined between serum PON1 activity and serum HDL levels (r = 0.935, P ˂ 0.001).

## 4. Discussion

The condition develops in case of an imbalance between oxidative stress, reactive oxygen species (ROS), and detoxifying systems. ROS are a heterogeneous group containing highly reactive ions and molecules derived from oxygen, such as superoxide anion (O2–), hydroxyl radical (OH_), hydrogen peroxides (H2O2), and singlet oxygen(O2–) [11]. ROS are detoxified by antioxidants such as vitamins C and E and enzymes such as paraoxonases (PONs). Increased ROS in the body results in damage to lipids, nucleic acids, and proteins. Rising ROS leads to DNA damage, changes in transcription and replication, induction of signaling transduction pathways, and genomic instability, the basis of carcinogenesis. Insufficiency in cellular antioxidant defense systems or increased ROS production results in increased oxidative stress and may play a role in the development of several types of cancer, including PCa. Studies have revealed that oxidative stress also affects the development and progression of PCa [12]. The present study compared paraoxonase (PON1, PON3), PON1/HDL, triglyceride, HDL, LDL, and MDA levels in patients with PCa among individuals with BPH, undergoing RARLP, with no biochemical recurrence and a control group. In addition, the levels of these enzymes in the postoperative first month after application of RALRP due to PCa and preoperative serum levels were also compared.

Paraoxonases are antioxidant enzymes that increase the hydrolysis of oxidized phospholipids and regulate oxidative damage, lipid peroxidation, the immune system, xenobiotic metabolism, and cell proliferation. Although the majority of paraoxonase enzyme functions involve paraoxonase and arylesterase activities, these enzymes are primary lactonases. PON3 exhibits no paraoxonase activity and very little arylesterase activity but has a greater lactonase activity than PON1 [13]. All three PON enzymes regulate lipid peroxides in low- and high-density lipoproteins. Paraoxonases are some of the main antioxidants that limit the accumulation of oxidized phospholipids in plasma lipoproteins [13]. HDL exhibits antioxidant properties by reducing oxidized lipids developing from cell membranes and other lipoproteins together with antioxidant enzymes such as apolipoproteins A-I and A-II and the PONs (PON1 and PON3) they contain [14]. Studies have also suggested that HDL plays an antitumoral role and have shown that HDL may be involved in the development and progression of PCa [15]. Plasma apoprotein A-I and HDL levels are inversely correlated with the risk of development of ovarian, breast, colonic, lung, and prostate cancers. Jafrive et al. [16] reported a reverse correlation between HDL levels and the risk of development and progression of PCa. Those authors showed that every 10 mg/dL increase in plasma HDL levels reduced the risk of cancer development by 36%. Yang et al. [17] reported that a 1 mg/dL decrease in HDL levels was associated with a 14% increased risk of cancer development. However, the relationship between HDL and cancer is still unclear because other studies have also reported no relationship or even a positive relationship between HDL levels and the risk of cancer [18]. No statistically significant difference was detected between the groups in terms of triglyceride, HDL, LDL, or cholesterol levels in the present research. This represents an original aspect of our study.

Arylesterase (ARE) and PON1 are serum esterases with powerful antioxidant properties and different activities of the same enzyme [19]. Eroglu et al. [20] observed no difference between PCa patient and control groups in terms of oxidative stress parameters and lipid parameters, although PON1 enzyme activity and LDL levels in PCa patients were higher/lower? than in the controls. In the present study, serum PON1 levels in patients with PCa were significantly lower compared to healthy individuals and patients with BPH. In addition, PON1 levels increased significantly in patients undergoing RALRP compared to the patient and control groups. This supports the idea of the elimination of malignant tissue in the body.

One metaanalysis reported significantly lower PON1 and ARE activities, in addition to serum HDL cholesterol, in patients with endometrium cancer, breast cancer, colorectal cancer, ovarian cancer, and stomach cancer [21]. Although research has also reported lower ARE enzyme activity in patients with bladder cancer than in a control group, the difference was not significant. In contrast, ARE activity remained normal in patients with PCa, while PON1 activity increased [21]. This suggests that PON1 is an enzyme with different active regions responsible for different activities.

PON1/HDL ratios have been proposed as a better parameter than PON1 activity alone [22]. However, there are studies showing that the ARE/HDL ratio is more significant than ARE levels alone in patient groups following the application of an energy-restricted diet [23]. In order to investigate whether the decrease in serum PON1 activity caused a decrease in HDL levels or enzyme activity, we calculated the PON1/HDL ratio. We observed no significant difference between the patient and control groups but a positive correlation between PON1 and HDL.

The mechanism involved in the decrease in PON1 and ARE activities in PCa patients is still unclear. This may involve an increase in lipid peroxidation because studies have shown that oxidized lipids inhibit PON1 and ARE activities [24]. Low PON1 activity in PCa patients may be a result of high ROS activity in PCa. This supports the postoperative increase in PON1 values after surgery.

Since PON3 has been less researched than PON1, less is known about its physiological functions. PON3 appears to be more powerful than PON1 in protecting against the oxidative modification of LDL and PON3 concentrations in serum are half of those of PON1. Animal studies have shown that PON3 is a more effective oxidative stress inhibitor than PON1 [25]. A significant increase in PON3 concentrations has been reported in patients with chronic liver disease, HIV, and coronary and peripheral artery disease, although PON3 protein in HDL has been reported to decrease in autoimmune diseases such as lupus erythematosus and type 1 diabetes and in patients with subclinical atherosclerosis [26]. Studies have shown that like PON2, PON3 has an oncogenic role and that PON3 is expressed in tumors of the pancreas, lung, bladder, ovary, kidney, and prostate [27]. In the present study, PON3 concentrations in patients with PCa were significantly lower compared to healthy individuals and patients with BPH.

Merendino et al. [28] reported that MDA is a noninvasive oxidative stress biomarker and that MDA levels in patients with BPH exhibit a positive correlation with PSA. In another study, Oparinde et al. [29] described MDA, like PSA, as a capable biomarker in detecting PCa. No correlation was determined between MDA and PSA in the present study, but a statistically significant positive correlation was observed between MDA and age.

MDA is a good marker of lipid peroxidation and a noninvasive biomarker of oxidative stress frequently used in the clinical setting to investigate free radical-mediated physiological and pathological conditions. MDA exhibits high cytotoxicity and an inhibitory effect on protective enzymes. It is therefore thought to be a tumor promoter and carcinogenic agent. MDA levels were significantly higher in patients with PCa compared to healthy controls and patients with BPH, suggesting that lipid peroxidation also increases in patients with PCa. In addition, we determined a statistically significant decrease in MDA levels in the postoperative first month in patients undergoing surgery due to PCa.

PON1 and PON3 are well known to exhibit antioxidant functions but differences in the activity and localization of these enzymes suggest that their functions also differ. A decrease in PON activity leads to a decrease in its antioxidant properties and increases exposure to oxidative stress. This situation then results in an increase in oxidative stress markers such as MDA. Serum PON3 decreased significantly in the PCa patients in this study compared to the control group. This was also accompanied by decreased PON1 concentrations and increased MDA activity.

Ahmed et al. [30] observed lower PON/HDL and PON/ARE ratios in patients with colorectal cancer compared to healthy controls and reported that the ratios returned to normal levels one month after surgery. Another study reported increased PON1 and ARE activity compared to preoperative levels in patients with papillary thyroid cancer and undergoing total thyroidectomy [31]. In the present study, PON1, MDA, and PON3 enzyme activities were investigated one month after surgical tumor excision. We observed increases in PON1 and PON3 levels and a decrease in MDA. This suggests that surgical tumor excision concluded with the removal of free radicals and a decrease in oxidative stress. However, the markers that are studied are found in many tissues in the human body and lack of tissue specificity restricts their use.

## 5. Conclusion

Changes in enzyme levels and pathways regulating oxidative stress and an increase in oxidative stress can lead to malignant and benign diseases of the prostate. Surgical excision of malignant tissue in PCa reduced oxidative stress. This suggests that the determination of standardized PON1, PON3, and MDA levels may be a useful biomarker in predicting recurrence following cancer surgery. Further randomized controlled studies on the subject are needed. We believe that the present study will also make a valuable contribution to the existing literature.

## Informed Consent

All procedures performed in studies involving human participants were in accordance with the ethical standards of the institutional and/or national research committee and with the 1964 Helsinki Declaration and its later amendments or comparable ethical standards. The study protocol (2018/18-169) was approved by the ethics committee of the Health Sciences University Erzurum Regional Education and Research Hospital. Informed consent was obtained from all individual participants included in the study.
